# The Influence of Space Transformation of Land Use on Function Transformation and the Regional Differences in Shaanxi Province

**DOI:** 10.3390/ijerph191811793

**Published:** 2022-09-19

**Authors:** Yaodan Zhang, Fei Li, Kai Li, Laiding Sun, Haijuan Yang

**Affiliations:** 1 College of Urban and Environmental Science, Northwest University, Xi’an 710127, China; 2 College of Natural Resources and Environment, Northwest A&F University, Xianyang 712100, China

**Keywords:** driving mechanism, function transformation, land use, space transformation, set pair analysis

## Abstract

The development of economy and urbanization promotes the transformation of land use both in space and function. Most existing research perspectives focus only on the transformation of space or function, and analysis of the mutual feedback mechanism between space transformation and function transformation is not deep enough. Therefore, this study constructed a diagnostic method for land use space transformation and function transformation and explored the mutual feedback mechanism between space transformation and functional transformation. The purpose is to deepen the research of land system science, provide a new method for diagnosing the space transformation and function transformation of land use, and clarify the driving mechanism of space transformation on function transformation as well as the mutual feedback mechanism of both. The research results showed that: (1) From 1980 to 2000, the space transformation of land use in the Northern Shaanxi Plateau did not happen, but the degree of trade-off between functions increased, resulting in the occurrence of unsustainable function transformation; the Guanzhong Plain showed a sustainable space transformation during the study period, but the land use functions did not transform; the function transformation and space transformation of the Qinba Mountain area happened in the same direction, and both tended to be sustainable; (2) From 2000 to 2018, the space transformation and function transformation of the Northern Shaanxi Plateau were in opposite directions; the land use space in the Qinba Mountain area was in a state of fluctuation and had not undergone transition, but the land use functions were in an unsustainable transition state; and (3) The function transformation in Shaanxi Province was deeply affected by factors at the policy and cultural levels and the strengthening of its own anti-interference ability, resulting in different performances of space transformation in different regions in different periods. Therefore, Shaanxi Province should rationally plan land resources, coordinate the relationship between space transformation and function transformation, and offer positive feedback to function transformation through sustainable space transformation. Meanwhile, it is necessary to prudently determine the regional land use model according to regional differences.

## 1. Introduction

Land serves as the social and material basis for human survival and development [1]. For these reasons, land should be preserved and restored from any form of anthropogenic pressure [2,3,4]. With the rapid development of society and economy, regional land use patterns have undergone a change in the long process, resulting in land use transition [5]. Since Long Hualou [6] introduced the concept of “land use transformation” into China in the early 21st century, many scholars have carried out significant and relevant research [7], including research frameworks [8], driving mechanisms [9], and the effects of land use transition [10] based on China’s national situation. Land use transformation includes two forms: land use space transformation and function transformation [11]. Space transformation can be diagnosed from the perspective of changes in land use quantity, landscape pattern, and management pattern. Function transformation refers to the changes in different combinations of land use functions [8]. The diagnostic criteria for function transformation of land use can be constructed from the perspective of externalities and policy development [7]. Some scholars have pointed out that changes in the spatial form of land use lead to reorganization of and change in the function of the land system and ultimately results in the completion of the function transformation of land use [12]. At present, many scholars have carried out a lot of research on a single level of space transformation or function transformation [12,13,14,15], but research on the mutual feedback mechanism of both is not deep enough. It is difficult to satisfy the development needs of the new era by independent research on the land use space transformation and function transformation. Therefore, the realization of sustainable development goals requires the rational allocation of both space transformation and function transformation of land use [16].

Specifically, Chinese scholars mainly combined Chinese characteristics and carried out a series of studies from various perspectives, such as the theory of land use transformation, social environment effects, and land function changes [17,18]. Foreign scholars focus more on the use of qualitative and quantitative research methods to explore the relationship between globalization and land use transition, environmental impact, and social-ecological effects [19,20]. In general, scholars comprehensively use qualitative and quantitative analysis methods to conduct more research on space transformation and function transformation from different perspectives [21,22,23,24], but most of them only focus on a single research topic to analyze their respective characteristics and different socio-economic effects. However, according to the theory of sustainable development, the transformation of land use in the new era should be described from two aspects: space transformation and function transformation of land use [11]. In contrast, based on the development requirements of the new era, research on the combination of space transformation and function transformation to explore its mutual mechanism and optimal control path is relatively weak. Moreover, land use transformation is the result of the long-term evolution trend of land use, which needs to judge whether land use transformation occurs according to the land use change in a long period of time [8]. It is only through the change of land use quantity, structure, operation mode, and function in a short period of time that whether land use has changed can be diagnosed, which confuses the judgment method of land use change and land use transformation [5]. From the perspective of the diagnostic methods of space transformation, such research methods are adopted as information entropy of land use structure [25], spatial lag model (SLM) [26] and spatial error model (SEM) [27], but these methods do not make quantitative and directional judgments on space transformation of land use. The function transformation diagnosis of land use is mostly to construct the evaluation index system of administrative regions. Most of them use comprehensive index method [28,29], improved TOPSIS [30,31], and other methods to measure the functional index of land use. However, such evaluation methods neglect the spatial heterogeneity of functions and the spatial correlation between different functions, and there is no quantitative criterion for judging the transformation [9]. Therefore, it is urgent to establish a quantitative and directional diagnosis model of land use space and function transformation to systematically analyze the driving mechanism and correlation mechanism of land use space transformation on function transformation.

Some scholars point out that the demand of land use value subjects for land use function transformation is the driving factor of land use transformation, and also say that there is a driving mechanism of land use space transformation to function transformation [11]. The development of social economy and the intensification of land use lead to the space transformation of land use, thereby reconstructing the quantity and pattern of land functions and promoting the occurrence of function transformation [32]. For the study of the impact of land use space transformation on function transformation, some scholars construct a comprehensive evaluation system of land use space-function transformation to quantify the impact of the combined effects of the two types of transformation on land use efficiency [33], but the deep driving mechanism of space transformation on function transformation has not been clearly revealed [34]. Therefore, focusing on the impact of space transformation on function transformation is the key to the current and future research on land use transformation.

Shaanxi Province is located on the Loess Plateau, which is one of the regions with the most serious soil erosion and the most fragile ecological environment in the world. Since the reform and opening up initiative, human activities, such as deforestation and grass cultivation, cultivated land reclamation, and urban expansion, have reconstructed the land use space, which in turn has led to the occurrence of function transformation. In addition, deeper factors, such as culture, system and concept, also lead to the occurrence of functional transformation of land use. Therefore, this research selects Shaanxi Province as the case study, we assume that the space transformation of land use has an ideal driving mechanism for function transformation. By constructing a quantitative and directional diagnosis model of space transformation and function transformation of land use, we systematically analyze the characteristics, internal factors and regional differences of land use transformation in Shaanxi Province, aiming to solve the following problems:

(a). What are the characteristics of space and function transformation of land use in different regions of Shaanxi Province?

(b). What is the deep driving mechanism of land use space transformation on function transformation?

The research results can enrich the related system of land use science research, expand the analysis ideas of land use transformation, guide the rational planning and management of land resources in Shaanxi Province, and provide reference for coordinating the relationship between land use, environmental protection, and grain security.

## 2. Material and Methods

### 2.1. Study Area

**Figure 1:**
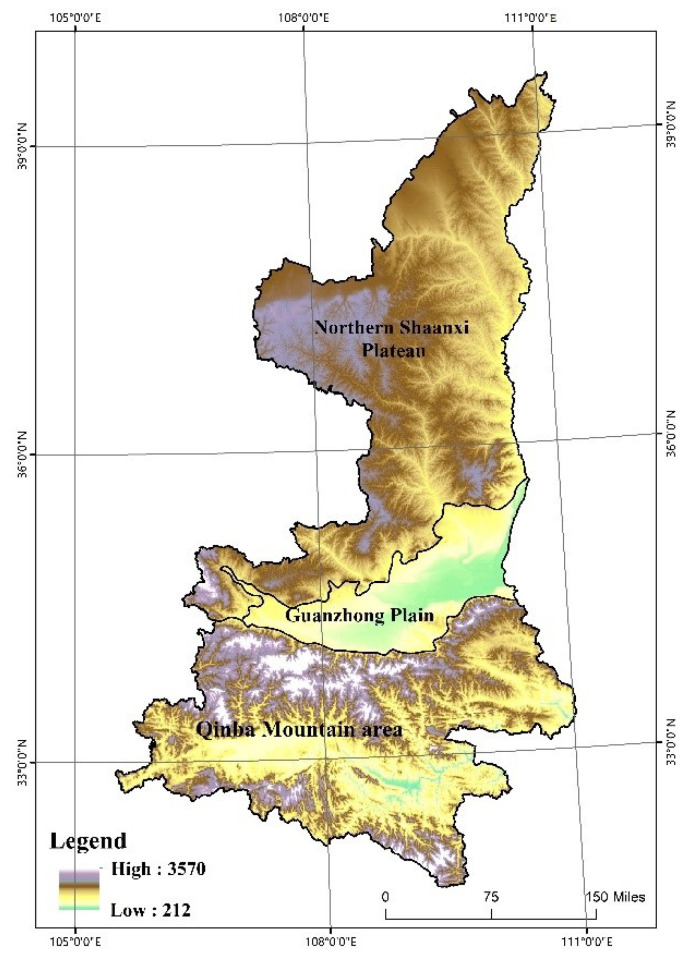
Study area map.

Shaanxi Province (105°29′ E~111°15′ E, 31°42′ N~39°35′ N) is located in Northwest China, in the middle reaches of the Yellow River Basin (Figure 1). The terrain is high in the north and south and low in the middle, with various types of land forms. According to the general landform type, it can be divided into three major regions from north to south, namely the Northern Shaanxi plateau, Guanzhong plain, and Qinba Mountain area. In recent years, under the guidance of the national “Western Development” strategy and the “One Belt, and One Road” initiative, Shaanxi Province has achieved rapid social and economic growth. However, growth is also achieved together with prominent ecological and environmental problems. The arable land is threatened, and land use has undergone an unsustainable transformation in space and function. Hence, in response to the major policies and guidelines of ecological civilization construction and cultivated land protection is proposed by the country. This research is based on the land use data of Shaanxi Province in the past 38 years since the reform and opening up (1978), in order to provide reference for the research framework of land use transformation and the rational development of land resources allocation in Shaanxi Province.

### 2.2. Data Sources

Land use transformation is the result of the long-term evolution trend of land use. It is necessary to judge whether land use transition has occurred according to the change of land use during a long period. Therefore, the time nodes are determined as the year of 1980, 2000, and 2018. The data types and sources are as follows (Table 1):

### 2.3. Diagnosis of Land Use Space Transformation

There are three forms of land use change: fluctuation, degradation, and optimization [35]. When the form of the land use system is fluctuating, there is no transition of land use. Unsustainable space transformation of land use occurs when system morphology is in a degraded state. Land use is manifested as a sustainable space transformation only when the system is in an optimized state. This study draws on the method proposed by Li et al. [35] to judge land use change patterns based on system vitality and resilience and conduct quantitative and directional diagnosis of land use space transformation.

**Figure 2:**
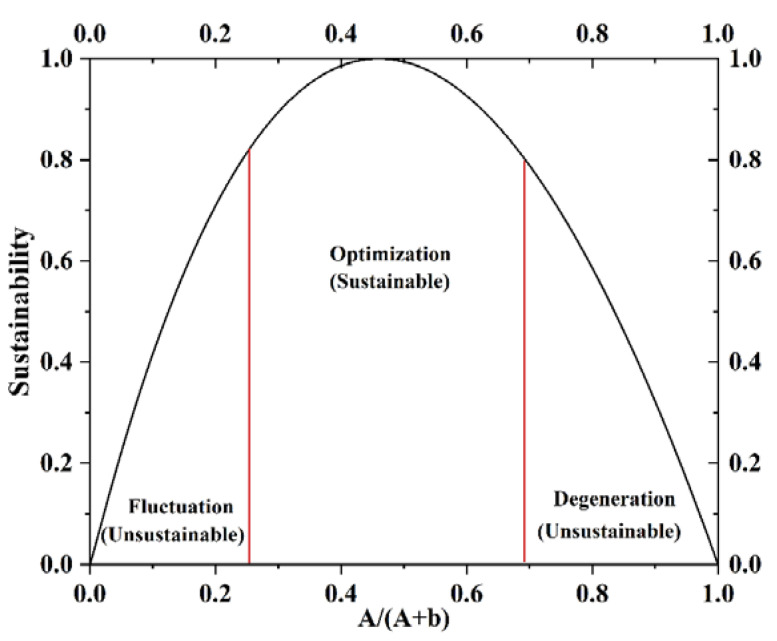
Space transformation form of land use.

System vitality (A) means the ability of the system to drive the effective ability to promote the spatial evolution of the system, and resilience (b) is the ability of the system to resist external disturbances [35] (Figure 2). Meanwhile, there is a trade-off between vitality and resilience. When the system vitality is too high, the land system is on the verge of collapse. When the system resilience is overly high, the vitality of the land system is inhibited, which is defined as a form of degradation. Only when the ratio of vitality and resilience of the system is appropriate, it indicates that the evolution of the land system is in an optimal state. β is an adjustment parameter. According to the calculation results of Ulanowicz et al. [36], β should be 1.288. It can be inferred that when *a* ∈ [0.2393, 0.6971], the land system is in an optimal state and sustainable space transformation occurs.

The calculation process is as follows:

(1) $$C=A+b=-\sum_{i,j}T_{ij}\log\left(\frac{T_{ij}}{TST}\right)$$

(2) $$A=\;\sum_{i,j}T_{ij}\log\left(\frac{T_{ij}TST}{T_{i}T_{j}}\right)$$

(3) $$b=-\sum_{i,j}T_{ij}\log\left(\frac{T_{ij}{}^{2}}{T_{i}T_{j}}\right)$$

(4) $$\mathrm{TST}=\sum_{i,j}T_{ij}\;T_{i}=\;\sum_{j}T_{ij}\;T_{j}=\sum_{i}T_{ij}$$

(5) $$S=-\frac{e}{\log\left(e\right)}a^{\beta }\;\log a^{\beta }$$

(6) a =A/C=A/(A+b)

In the formula, TST represents the total land area that has been transformed, *T_i_* represents the area of land converted from type *i* to other types of land, and *T_j_* represents the area of land converted to type *j* of land. *T_ij_* represents the land area converted between class *i* and class *j*.

### 2.4. Land Use Function Assessment

Due to the fragile ecology and poor habitat quality of Shaanxi Province, the main characteristics of its land use changes in recent years mark the conversion of farmland to forests and the expansion of construction land. Although returning farmland to forests increases ecological services, it then reduces the area of arable land and affected grain production. Other land use functions (such as employment, housing security, and economic development functions) are equally important, but these functions are more affected by social and economic development. In addition, these functions vary greatly in space and are mainly concentrated on construction land, which accounts for a small proportion of land use in Shaanxi Province, accounting for only 2.7% of the province’s land area in 2018. Therefore, changes in functions, such as employment, housing security, and promoting economic development are mainly affected by social and economic development, rather than by the space transformation of land use. Based on the above considerations, this paper focuses on two functions, ecological services and grain production.

(1) Calculation of ecosystem service value (ESV):

Ecological function is characterized by ecosystem service value. The evaluation method of Xie et al. [37,38] is to define the annual natural output of farmland with an average yield of 1hm^2^ nationwide as the economic value of an equivalent factor. According to the calculation, its value roughly equals to 1/7 of the value of grain yield per unit. Because the problems involved are more complicated when measuring the ecosystem service value of construction land. It is difficult to obtain evaluation data as well. Hence, this paper defines the ecosystem service value of construction land as 0 during the evaluation. In addition, the ecosystem service value varies during different periods and different social stages, but in order to better explore the changes of ecosystem service value caused by the single factor of land use space transformation, this paper fixes the social development stage coefficient. Based on the economic price, average price, biomass and other data of grain production in Shaanxi Province in the year of 1980, 2000, and 2018, the equivalent factor is corrected for temperature and precipitation to apply to the actual situation of Shaanxi Province, and the study obtains the value of a single ecosystem service value equivalent factor. The amount of value is 790.32 yuan/hm^2^.

Adopting the ecosystem service value evaluation model, the total ecosystem service value of each grid of 1 km × 1 km in Shaanxi Province is calculated. The calculation formula is as follows:

(7) $$\mathrm{ESV}=\sum_{i=1}^{m}\sum_{j=1}^{n}M_{ij}\;\times \;E_{a}\;\times \;C_{t}$$

In the formula, ESV is the total value of ecosystem services in the study area (100 million yuan), and $M_{ij}$ is the numerical table of quantitative factors corresponding to the jth ecosystem service function of the ith type of land; ***m*** is the number of land use types; ***n*** is the number of ecosystem service functions, $E_{a}$ represents the economic value of grain output per unit area, $C_{t}$ is the revised social development stage coefficient.

(2) Calculation of grain production potential (GAEZ model):

The function of grain production is measured and characterized by the potential of grain production. In order to quantitatively estimate the potential yield of grain in a certain region, the Food and Agriculture Organization of the United Nations (FAO) and the International Institute for Applied Systems Analysis (IIASA) have developed a global agro-ecological zoning model (GAEZ) [39,40,41]. The GAEZ model first estimates the climatic suitability of planting a certain crop according to the climatic conditions and then corrects the crop photosynthetic production potential (light limitation only)–light temperature production potential (light and temperature limitation)–light and warm water production potential (light, temperature and water limitations)–climate production potential (agroclimatic disaster limitations)–crop production potential (soil and various management measures limitations) in a gradual manner [42]. The model’s estimates of grain production potential consider irrigation and raining conditions, respectively [43,44]. This calculation method was in line with agricultural production, so this paper directly uses the grain production potential under rain-fed conditions to evaluate the actual situation of grain production in Shaanxi Province.

(8) $$yield_{actual}=yield_{total}\;\times \;lu\%$$

(9) $$yield_{total}=yield_{rain-fed}\;\times \;\left(1-i\right)+yield_{irrigated}\;\times \;i$$

where $yield_{actual}$ represents the grain production potential under different scenarios, unit: kg·hm^−2^·arl, lu% represents the proportion of arable land in the pixel; $yield_{total}$, $yield_{rain-fed}$, $yield_{irrigated}$ represent the total grain production potential, the grain production potential under rainfed conditions, and the grain production potential under irrigated conditions, respectively; *i* represents the irrigation rate.

### 2.5. Functional Transformation Diagnosis of Land Use

SPA-MI model: Anselin [45] proposed bivariate spatial autocorrelation in 1995, which was a concept to explore the correlation between a data attribute value in the same space and another data attribute value adjacent to each other. In this paper, the global Moran’s I index and sets pair analysis were integrated, and the SPA-MI model [46] was constructed to calculate the correlation between functions to diagnose whether the land use function is transformed.

(10) $$\mu =\;\frac{S}{N}\;+\frac{F}{N}I-\frac{P}{N}$$

Among them, μ is the degree of correlation, and $\frac{S}{N}$, $\frac{F}{N}$, and $\frac{P}{N}$ represent the degree of identity, difference, and opposition of these two functions, respectively. A bivariate global autocorrelation analysis is performed on these two land functions, “I” is Moran’s I index, and the total number of “high-high” and “low-low” is “*S*”. The total number of “high-low” and “low-high” is “*P*”; the number of “insignificant” is “*F*”; *N* = *S* + *F* + *P*.

The relationship between the two functions is divided into the following four types (Table 2): trade-off, partial trade-off, partial synergy, and synergy. According to the integration result, it can be concluded that if the degree of association is μ∈[−1, −0.25), the relationship between the two functions is determined to be a “trade-off”. Similarly, if it is μ∈[−0.25, 0), the relationship between functions is a “partial trade-off”; if it is μ∈(0, 0.32], it is “partial synergy”; μ∈(0.32, 1], means “synergy” relationship [47], in order to judge whether the land use function has transformed (for example, if the relationship between the two functions from period A to period B changes from partial trade-off to trade-off, an unsustainable transition will occur).

In order to further explore the spatial agglomeration characteristics of land use functions, this study explores the spatial trade-off transition characteristics of the two functions based on bivariate local spatial autocorrelation [47]. The bivariate global Moran’s I index formula is:

(11) $$I=\frac{n\sum_{i}^{n}\sum_{j}^{n}W_{ij}Q_{k}^{i}Q_{k}^{j}}{\left(n-1\right)\sum_{i}^{n}\sum_{j}^{n}W_{ij}}$$

A bivariate local Moran’s I is defined as:

(12) $$\mathrm{Is}=Q_{k}^{i}\sum_{j=1}^{n}W_{ij}Q_{l}^{j}$$

In the formula $Q_{k}^{i}$ = $\frac{X_{k}^{i}-X_{k}}{\partial_{k}},\;Q_{l}^{j}$ = $\;\frac{X_{l}^{j}-X_{l}}{\partial_{l}};\;$ *n* is the number of space units; $X_{k}^{i}$ is the space value of the unit *i* attribute *k*; $X_{k},\;\;X_{l}$ is the average value of attributes *k*, *l*; $\partial_{k},\;\partial_{l}$ are the variances of attributes *k*, *l*; $W_{ij}$ is a weight matrix that measures the adjacency relationship between spatial units.

## 3. Results

### 3.1. Characteristics and Regional Differences of Land Use Spatial Transformation

**Figure 3:**
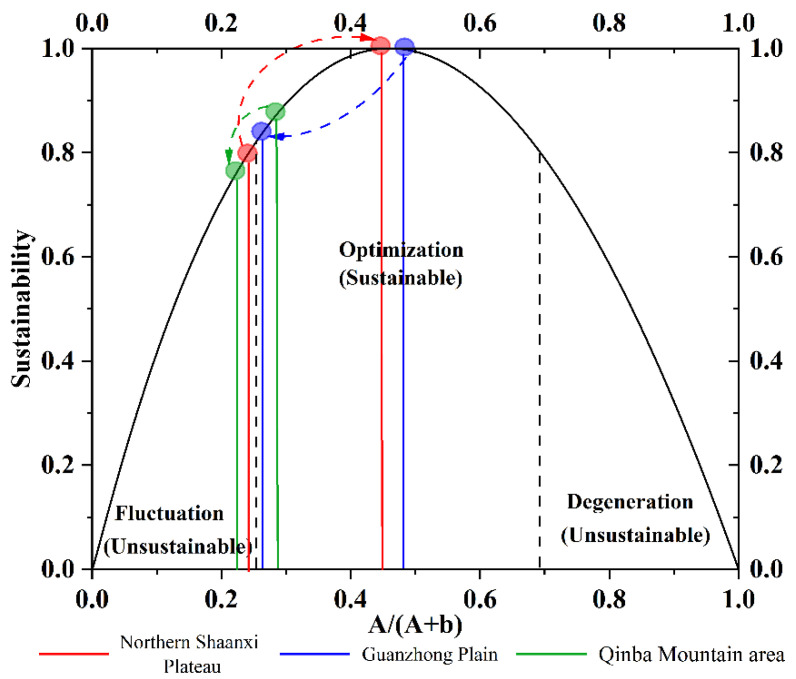
Diagnosis of Spatial Transformation of Land Use in Three Major Regions of Shaanxi Province from 1980 to 2018. Note: The direction of the arrow represents the transition from the 1980–2000 stage to 2000–2018 in the three major regional sustainability of Shaanxi Province.

**Figure 4:**
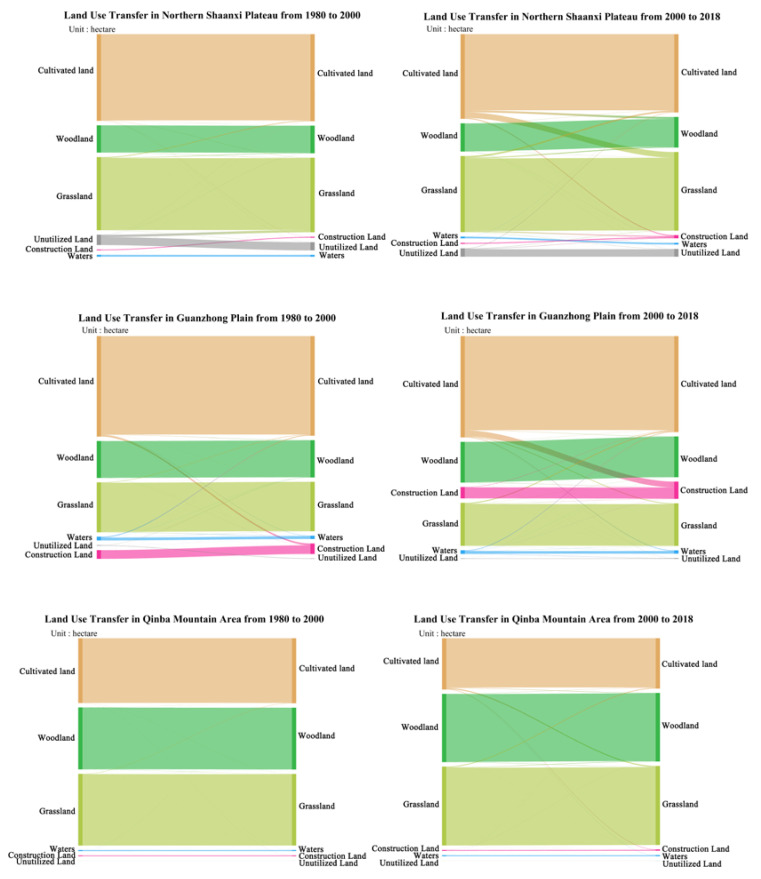
Land use transfer in three geographical units from 1980 to 2018.

Based on the land use transformation matrix of 1980–2000 and 2000–2018, this paper calculates the spatial sustainability index of land use in the three major regions of Shaanxi Province in different periods(Table 3, Figure 3), and draws the land use transfer map and the dominant transfer type table of the three geographical units in Shaanxi Province (Table 4, Figure 4) so as to explore the space transformation of land use in Shaanxi Province more intuitively. The results show that the spatial pattern of land use in Shaanxi Province is basically stable during the study period, but the sustainability of land system evolution fluctuates over time. The increase of grassland area results in the enhancement of ecological functions, which promotes the sustainability of the land system.

From 1980 to 2000, sustainable space transformation occurs in Guanzhong Plain and Qinba Mountain area. The land system in the Northern Shaanxi Plateau is fluctuating with a sustainability of 0.7884 (Table 3, Figure 3), which is lower than that of the Guanzhong Plain and Qinba Mountain area in the same period. The main reason is that the ecological protection of the Northern Shaanxi Plateau increased in the past 20 years, and the area of unused land converted to grassland reached 13.01 million hectares (Figure 4, Table 4), which improved the resilience of the land system. At this stage, the land system in the Guanzhong Plain was in an optimized state and the sustainability was stabled above 0.8 (Sustainability: 0.9967). Although the economy of the Guanzhong Plain developed rapidly during this period, 33,500 hectares of cultivated land is occupied by construction land, which provides vitality for the evolution of the system. However, due to the implementation of ecological protection policies, such as returning farmland to forests and protecting grasslands, a large amount of unused land is converted into forest land and water areas, and the vitality and resilience ratio of the land system is thus properly maintained, resulting in a sustainable space transformation of land use. Due to numerous natural forests and nature reserves, good ecological background and strong system resilience, sustainable space transformation of land use occurs in Qinba Mountain area.

From 2000 to 2018, the Northern Shaanxi Plateau and the Guanzhong Plain showed sustainable space transformation. The sustainability of the Northern Shaanxi Plateau reached 0.998, mainly due to the implementation of the pilot project of returning farmland to forest in 1999. Nearly 3147 million hectares of cultivated land were transferred to grassland, which provided a marginal contribution to its sustainable space transformation. The land system of Guanzhong Plain was in an optimized state, and the land use had undergone a sustainable space transformation. Although 1,136,000 hectares of cultivated land converted to construction land, the “centripetal force” of the Guanzhong urban agglomeration made the construction land expand in a cluster manner. Meanwhile, the construction land mainly occupied cultivated land, which had a weak direct impact on ecological land. Therefore, land use still showed a sustainable space transformation.

To sum up, the space transformation of land use in different regions of Shaanxi Province in different periods showed different characteristics.

### 3.2. Function Transformation Characteristics of Land Use and Regional Differences

Based on the SPA-MI model, the correlation between ecological function and grain production function in these three regions from 1980 to 2018 is calculated and the relationship between them is diagnosed (Table 5). On this basis, it is also diagnosed whether there is a positive function transformation of land use.

From 1980 to 2000, there was an unsustainable function transformation in the Northern Shaanxi Plateau, but the Qinba Mountain aera showed a sustainable function transformation on the contrary. During this period, the correlation between the functions of the Northern Shaanxi Plateau decreased by 0.115 (Table 5). The relationship between the two functions gradually changed from synergy in 1980 to partial synergy in 2000. Since the heavy industrial economy of the Northern Shaanxi Plateau accounted for 1/4 of the total economic output of Shaanxi Province, the problem of land degradation caused by industrial pollution has caused great pressure on the already fragile ecological environment, resulting in the relationship between grain production function and ecological function from synergy to partial synergy, which led to the occurrence of unsustainable transformation of land functions. The correlation between the two functions in the Guanzhong Plain decreased by 0.062. Due to people’s weak awareness of ecological protection, a series of measures, such as cultivated land reclamation since the reform and opening up have made the ecological function and grain production function in a trade-off state. With the progress of people’s ideology and the guidance of ecological protection policies, the trade-off relationship between the two functions tended to ease, and the land use function underwent sustainable transformation.

From 2000 to 2018, the land use function of the Northern Shaanxi Plateau and the Qinba Mountain area had an unsustainable transformation, but there was no transformation in the Guanzhong Plain. The correlation between the two functions of the Northern Shaanxi Plateau decreased by 0.342, which was three times larger than that of the previous period. The reason may be that the comprehensive implementation of the policy of returning farmland to forest in the past two decades greatly reduced the cultivated land area [48], and the relationship between grain production function and ecological function changed from partial synergy to partial trade-off, leading to the occurrence of unsustainable function transformation. During this period, the effective implementation of ecological protection policies, such as ‘closing hills to forest, returning farmland to forest’ and the ‘decision on deepening reform and strict land management’ in 2004 made the trade-off increase by 0.035, and the trade-off between the two functions was eased. Since the reform and opening up initiative (1978), a number of township enterprises have emerged in Qinba Mountain Area, which has injected vitality into social and economic development. However, at the same time, a large number of natural forests and nature reserves have been destroyed, resulting in declining of ecological functions. In addition, the increase of rural population and the extensive use of pesticides led to an intensified trade-off between the two functions, resulting in the occurrence of unsustainable transformation.

**Figure 5:**
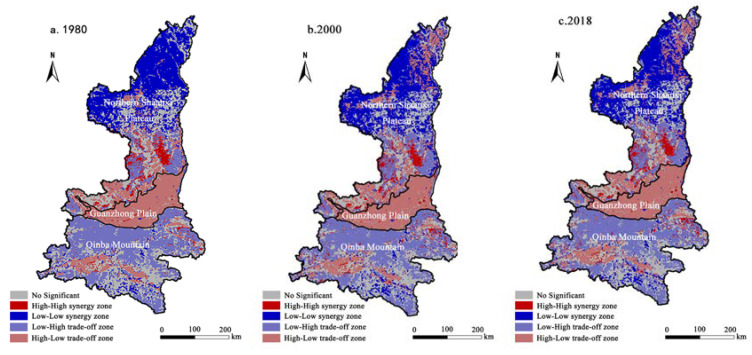
LISA agglomeration map of grain production function and ecological function in three major regions of Shaanxi Province from 1980 to 2018.

From the bivariate local LISA diagram, there exists large regional differences in the agglomeration between the spatial correlation among these three regional functions (Figure 5). The Northern Shaanxi Plateau is dominated by the low-low synergy area, and the high-high synergy area is distributed in groups in the central region with flat terrain suitable for farming. From 1980 to 2000, the “high-low trade-off area” in the northern part of the Northern Shaanxi Plateau expanded to a large extent, transforming unused land, such as wasteland, saline-alkali land, and marshland, into cultivated land through reclamation (for example, many large centralized farms were established in the Mu Us Desert region [49]), resulting in the transformation of unused land reaching 22,500 hectares. As a result, the function of grain production was improved, and the corresponding ecological function was lessened. The two were antagonistic, which provided a ‘marginal benefit’ for the unsustainable function transformation. Qinba Mountain aera was dominated by low-high trade-off areas, while high-low trade-off areas were scattered in the south.

In summary, there have been obvious differences in function transformation of land use and spatial agglomeration characteristics among the three regions in Shaanxi Province during this period. The policy, economic development, and urbanization expansion led to the spatial replacement of land use function, which became a deep driving factor for the trade-off between grain production function and ecological function and the transformation.

### 3.3. Study on the Driving Mechanism of Land Use Space Transformation on Function Transformation

Based on the change of ecosystem service value and grain production potential caused by land use transition, this paper analyzes the impact of land use space transformation on function transformation in three regions. The results show that the space transformation of land use in the three regions has a driving effect on function transformation.

The sustainability index of the Northern Shaanxi Plateau from 2000 to 2018 was 0.998, but the correlation between the two functions decreased by 0.342. Sustainable space transformation of land use occurred, but it was manifested as unsustainable function transformation (Table 6). As a result of the state’s most stringent cultivated land protection system to protect the red line in cultivated land in 2009, 4.28 million hectares of cultivated land were converted to woodland, grassland and water, making a substantial increase in the value of ecosystem services (Table 7 and Table 8). The rise in sustainability of land systems contributed to the occurrence of sustainable space transformation of land use. With the development of urbanization, the status of the secondary industry became increasingly prominent. The expansion of urban construction land continued to squeeze cultivated land, threatening the function of grain production. The conversion of 47,000 hectare cultivated land to construction land led to a decrease in grain production potential of 1119.48 kg/ha (Table 9). Then, in order to balance occupation and compensation, the corresponding quantity and land quality were compensated to protect the red line of cultivated land from not being crossed. Therefore, the trade-off between ecological function and grain production function was intensified, leading to the occurrence of unsustainable function transformation.

Sustainable space transformation of land use happened throughout the study period in Guanzhong Plain, while function transformation of land use did not occur. Due to the relatively advanced economy and technology in Guanzhong Plain, as well as the development guidance of building Guanzhong-Tianshui Economic Zone [49], the promotion of land use patterns, such as compound utilization and recycling, led the cultivated land in the Guanzhong Plain to be the main source of the transformation of construction land, resulting in a loss of 917.8949 million yuan in the value of ecosystem services. It is five times the loss of ecosystem service value caused by the conversion of forest land and grassland to construction land (Table 7). It can be seen that Guanzhong Plain occupies a large number of cultivated land in the process of economic development, and that the vitality of the land system was high. However, the occupation of ecological land is relatively small, and the direct interference of urbanization on ecological land is light. From the perspective of the internal relationship of land use function, the relationship between ecological function and grain production function has been in a trade-off state. The conversion of cultivated land to woodland, grassland, and water area results in a total decrease of 11,519.34 kg/ha in grain production potential. Therefore, the synergy between ecology and grain production function is stable, and land use function has not been transformed.

From 1980 to 2000, the transformation directions of space transformation and function transformation of land use in Qinba Mountain Area were consistent, with sustainable transformation occurring. During this period, due to the limitation of mountainous terrain conditions, economic development lagged. The growth of residential and industrial land was slow. Therefore, only 0.0004 million hectares of construction land occupied ecological land, resulting in the loss of ecosystem service value accounting for only 3% of the total loss. Moreover, the expansion of cultivated land did not threaten the ecological land. In addition, Qinba Mountain Area serves as the key area of ecological conversion of farmland and comprehensive management of small watershed in Shaanxi Province. Its natural conditions were relatively superior. Good resource endowments maintained the stability of the system and improved the anti-interference ability of the system. Therefore, land use underwent a sustainable space transformation.

Based on the above analysis, it is found that the space transformation of land use has a driving effect on function transformation, but the performance characteristics are different in various periods. The underlying reason for this result may be the influence of policy and cultural factors [8]. In 1980–2000, the space transformation of land use in the Qinba Mountainous Area, with less policies adopted and better retention of land environmental attributes, has an obvious driving effect on function transformation. The transformation direction is consistent. However, in the Northern Shaanxi Plateau and Guanzhong Plain, where human activities are more complex, the transformation of land use functions does not fully show the characteristics of changes in synchronization with the space transformation. Therefore, we conclude that spatial transformation is a deep driving mechanism affecting the path of land function reconstruction. At the same time, the transformation of land use function is also jointly driven by policies, culture, resource endowments, and its own development of the system.

## 4. Discussion

Land system is a mutual system of social economic system and ecological environment system. Driven by deep and surface factors, land use transformation leads to a series of social economic and ecological environment effects [50]. At present, the research on land use transformation mainly only focuses on one level of land use space transformation or function transformation [51,52]. By combining both transformations, the research on the driving mechanism based on the development requirements of the new era is relatively weak. Additionally, the mechanism of the mutual feedback relationship between the space transformation of land use and the transformation of function is not clear for the current theoretical research on land use transformation. Furthermore, the current diagnostic methods of land use space transformation and function transformation only stay in the level of land use quantity and structure change, lacking for quantitative and directional diagnosis [26,27,28,29]. Therefore, this paper constructed a diagnosis model of land use space transformation and function transformation to explain the mutual relationship and boosting mechanism between them.

**Figure 6:**
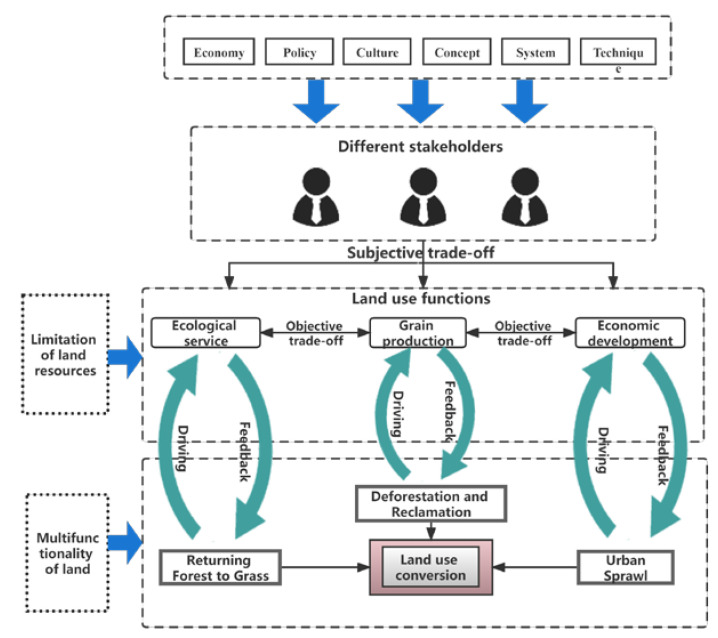
Research framework of transmission mechanism of land use space transformation on function transformation.

Due to the objective fact of the multi-functionality of land use, the transformation of the spatial form will not only produce social economic and ecological environmental effects, but also lead to the transformation of land use functions. The change of land use functions will also develop with the spatial form of land use, inducing space transformation [53,54]. In order to explore the driving mechanism of the space transformation of land use on the function transformation, this paper constructs the “Research Framework of the Transmission Mechanism of the Impact of space transformation on function transformation” (Figure 6).

The mechanism of land use transformation can be explained from two aspects: “socio-ecological feedback” and “socio-economic dynamics”. The basis of “socio-ecological feedback” is the limited land resources [8]. The excessive consumption of land resources by social development leads to the shrinkage of ecological service space, which feds back into the social and economic system and threatens the survival and development of human beings, and finally forced human beings to take measures to protect and restore ecological service space. The transformation of land use occurred [55]. “Socio-economic dynamics” base on the versatility of land. Land functions are transferred by people’s values [10]. When using land, various stakeholders will give land what functions and roles they should undertake according to their own needs. Private and public products and services provided by different land use methods are different. Therefore, on limited land, there will be subjective trade-offs between different stakeholders on land use functions. With the growth of social economy, the land rent and output of different land use types will change. When the land rent or opportunity cost of a certain land is higher than its output, the stakeholders will change its original land use type, leading to land use transformation [20,56]. It can be seen from the mechanisms of ‘social-ecological feedback’ and ‘social-economic dynamics’ that the group response of land system change is more important than the individual response in driving land use transformation [8,20]. The driving force of group response to land use transformation includes: infrastructure construction, urban expansion, agricultural land expansion, forest harvesting, returning farmland to forests and grasslands and other obvious factors; deep rooted factors lie in the changes of system, policy, economy, population, location, culture, concept and technology. The deep driving factors act on those that are obvious through the “socio-ecological feedback” mechanism to form a cascade effect, which promotes the space transformation of land use. The deep driving factors form a cascade driving effect along the “socio-economic dynamics” mechanism, which constitutes the functional transformation of land use and counteracts the space transformation of land use [8,57].

Institutional change and policy adjustment are deep driving factors that are major influences in the process of regional land use transition [58,59]. Regional land use transition under the guidance of policy is the result of the combined effect of ‘social-ecological feedback’ path under the guidance of policy and ‘social-economic dynamic’ induced path under the influence of policy. The former determines the direction of regional land use transition, and the latter determines the speed and intensity of regional land use transition [60]. The process of economic development and economic modernization has brought about the intensive use of land, which can enhance the ecological and environmental protection awareness of different stakeholders and change their land use behavior. Government policies can directly affect the degree of protection of different ecosystem services by stakeholders, such as returning farmland to forest policy, ecological compensation mechanism, nature reserve planning, etc., which can effectively tackle the environmental problems in ecologically fragile areas and maximize the benefits of regional ecosystem services [61]. Therefore, government policies and management of stakeholders have also become the focus of the driving mechanism of land use function transformation.

Since the reform and opening up initiative, the urbanization and industrialization development strategy of Shaanxi Province has continuously promoted the spatial integration and flow of social and economic development factors. Although it has improved the level of social and economic development as a whole, it also squeezes the ecological and grain production areas, resulting in land use moving from sustainable to unsustainable. However, due to policy intervention, the speed of unsustainable transformation of land use space has slowed and even transformed into sustainable transformation. For example, the implementation of the Northern Shaanxi Plateau Ecological Project has a significant effect on the improvement of resources and environment. The sustainability increased from 0.7884 in 1980–2000 to 0.998 in 2000–2018, promoting the transition of land use to sustainable space. As the quantity and structure of land use are in the process of continuous flow, growth and reorganization, the conversion of land use types promotes the function transformation. The land with low land rent output capacity (such as forest land, grassland, cultivated land) has the power to transfer to high land rent output capacity (such as urban construction land) [8]. Therefore, a series of human behavior driven by multiple factors, such as returning forest to grassland, deforestation, and reclamation. The occupation of cultivated land by construction land reconstructs the land use functions from quantity and quality, so that the land use functions are transformed again, providing power for the function transformation of the next stage [62,63]. The interaction between space transformation and function transformation of land use has a profound impact on the realization of grain security, ecological protection and high-quality development.

Based on the characteristics of spacel transformation and function transformation of land use in Shaanxi Province since the reform and opening up, this paper expands the conceptual connotation of land use transformation, integrated the land use transformation mechanism (“social-ecological feedback” and “social-economic dynamics”), constructs the transmission path analysis framework of land use space transformation-function transformation, and explains the coupling relationship and boosting mechanism between the two. The paper aims to propose corresponding optimization paths for the overall direction of land use transformation in the future and provides theoretical reference for local governments at all levels to carry out land use transformation research and practice.

## 5. Conclusions

Based on the impact of land use space transformation on function transformation, this study establishes a method of land use space transformation based on vitality and toughness, an integrated analysis and bivariate autocorrelation function transformation method to study the transition mechanism of land use space transformation on function transformation in three major regions of Shaanxi Province since the reform and opening up. From the perspective of land use space transformation, the results show: the characteristics of land use space transformation in different periods and regions are significantly different. During the study period, land use in the Northern Shaanxi Plateau is transitioning to a sustainable space transformation. The Guanzhong Plain maintained a sustainable spatial transition of land use, and the sustainable spatial transition in the Qinba Mountain area gradually slows down. Through the analysis of the existing research, it can be seen that the research conclusion of identifying the space transformation of land use based on vitality and resilience was credible [35].

From the perspective of land use function transformation: it can be concluded that the three major regions of Shaanxi Province vary greatly in land use function transformation and space agglomeration characteristics. During the study period, the trade-off relationship of the Northern Shaanxi Plateau shows a “stepped” evolution, reflecting an unsustainable transformation as a whole. The Guanzhong Plain does not undergo transformation. The land use function of the Qinba Mountain area tends to undergo an unsustainable transformation.

The space transformation of land use in Shaanxi Province has a driving effect on the function transformation, but the performance characteristics vary in different periods. The main reason for the result is that it is influenced by factors at the policy and cultural levels, as well as its own internal reinforcement. The external and internal factors lead to different performance characteristics of driving space transformation to function transformation.

## Figures and Tables

**Table 1:** Data types and sources.

Data Type	Key Indicators	Data Source and Processing
Land use data	Including 6 first-level land use types (Arable land, Forest land, Grassland, Water area, Construction land, and Unused land)	The three-phase land use data in 1980, 2000, and 2018 were obtained from the remote sensing monitoring data of the Resource and Environmental Science Data Center of the Chinese Academy of Sciences (http://www.resde.cn, accessed on 25 December 2021), with a resolution of 30 m.
Terrain data	DEM	The digital elevation model (DEM) data were obtained from the Data Center of Resource and Environmental Sciences, Chinese Academy of Sciences, with a resolution of 90 m.
Soil data	Soil type, structure, etc.	Sourced from the “China Soil Dataset (v1.1) based on the World Soil Database (HWSD)” (http://westdc.westgis.ac.cn, accessed on 10 January 2022) by the Cold and Arid Regions Science Data Center, with a resolution of 1km.
Meteorological data	It includes daily and monthly data, such as precipitation, temperature, radiation, and evapotranspiration in 1990, 2000, and 2018.	From China Meteorological Science Data Sharing Network (http://cdc.cma.gov.cn, accessed on 10 January 2022), and it is interpolated by Anusplin.
Socioeconomic data	Per capita GDP of the whole country and Shaanxi Province in 1980, 2000, and 2018, Engel coefficient of urban and rural areas, economic price of grain output, average price, etc.	Sourced from the National Bureau of Statistics of China and the Statistical Yearbook of Shaanxi Province.

**Table 2:** Classification of Land Use Function Balance.

Relevance (μ)	Tradeoffs
[−1, −0.25)	trade-off
[−0.25, 0)	partial trade-off
(0, 0.32]	partial synergy
(0.32, 1]	synergy

**Table 3:** Space Transformation Types of Land Use in Three Major Regions of Shaanxi Province from 1980 to 2018.

Area	Period	1980–2000	2000–2018
Northern Shaanxi Plateau	α	0.2366	0.4376
	S	0.7884	0.9980
	The spatial evolution form of the land system	Fluctuation	Optimization
	Types of land use transitions	Not transformed	Sustainable transformation
Guanzhong Plain	α	0.4890	0.2553
	S	0.9967	0.8237
	The spatial evolution form of the land system	Optimization	Optimization
	Types of land use transitions	Sustainable transformation	Sustainable transformation
Qinba Mountain area	α	0.2907	0.2242
	S	0.8809	0.7631
	The spatial evolution form of the land system	Optimization	Fluctuation
	Types of land use transitions	Sustainable transformation	Not transformed

**Table 4:** Land use transfer types dominated by three geographical units.

	1980–2000	Area (10,000 Hectares)	2000–2018	Area (10,000 Hectares)
Northern Shaanxi Plateau	Unutilized land → Grassland	13.01	Cultivated land → Grassland	31.47
Guanzhong Plain	Cultivated land → Construction land	3.35	Cultivated land → Construction land	11.36
Qinba Mountain aera	Grassland → Cultivated land	1.38	Cultivated land → Grassland	4.81

**Table 5:** Types of Land Use Function Transformation in Three Major Regions of Shaanxi Province from 1980 to 2018.

Area		1980–2000	2000–2018
Northern Shaanxi Plateau	Changes in Correlation	−0.115	−0.342
	Weighing Relationship Changes	synergy—partial synergy	partial synergy—partial trade-off
	Type of Transformation	Unsustainable transformation	Unsustainable transformation
Guanzhong Plain	Changes in Correlation	−0.062	0.035
	Weighing Relationship Changes	partial trade-off-partial trade-off	partial trade-off-partial trade-off
	Type of Transformation	Untransformed	Untransformed
Qinba Mountain aera	Changes in Correlation	0.057	−0.021
	Weighing Relationship Changes	trade-off—partial trade-off	partial trade-off—trade-off
	Type of Transformation	sustainable transition	Unsustainable transformation

**Table 6:** Comparison of Space Transformation and Function Transformation of Land Use in Three Major Regions of Shaanxi Province from 1980 to 2018.

Area	Period	Transition Type		Changes in Sustainability Index	Changes in Correlation
		Space Transformation	Function Transformation		
Northern Shaanxi Plateau	1980–2000	Untransformed	Unsustainable transformation	0.7884	−0.115
	2000–2018	Sustainable transformation	Unsustainable transformation	0.9980	−0.342
Guanzhong Plain	1980–2000	Sustainable transformation	Untransformed	0.9967	−0.062
	2000–2018	Sustainable transformation	Untransformed	0.8237	0.035
Qinba Mountain aera	1980–2000	Sustainable transformation	Sustainable transformation	0.8809	0.057
	2000–2018	Untransformed	Unsustainable transformation	0.7631	−0.021

**Table 7:** Changes of ecosystem service value caused by land use transformation in three geographical units from 1980 to 2000 (unit: 10,000 yuan).

1980		2000					
		Cultivated Land	Woodland	Grass Land	Waters	Construction Land	Unutilized Land
Northern Shaanxi Plateau	Cultivated land	0.00	9573.82	3323.94	4073.91	−4441.86	−2084.17
	Woodland	−5090.26	0.00	−16,097.70	136.28	−397.23	−429.91
	Grassland	−12,399.54	31,904.90	0.00	2178.00	−795.91	−6716.91
	Waters	−6065.71	−113.56	−5064.79	0.00	−63.32	−2270.98
	Construction Land	2.76	1.09	2.04	1.12	0.00	0.02
	Unutilized Land	2037.59	7700.94	105,689.94	115.82	−30.81	0.00
Guanzhong Plain	Cultivated land	0.00	9612.44	263.99	17,069.63	−20,887.76	−81.27
	Woodland	−376.27	0.00	−656.61	438.01	−3694.54	−77.43
	Grassland	−2685.25	947.73	0.00	19,159.12	−301.11	−94.44
	Waters	−49,564.04	−1708.56	−10,424.44	0.00	−423.64	−885.72
	Construction Land	360.89	3.78	201.94	1.11	0.00	0.00
	Unutilized Land	156.23	6711.60	0.09	2396.02	−2.08	0.00
Qinba Mountain aera	Cultivated land	0.00	2977.40	1187.50	2968.20	−1233.11	−0.06
	Woodland	−5926.78	0.00	−8625.80	176.99	−703.25	0.00
	Grassland	−4104.29	5114.22	0.00	2317.42	−2.70	0.00
	Waters	−6041.50	−152.29	−2003.73	0.00	−182.71	−0.01
	Construction Land	3.29	0.25	2.83	1.00	0.00	0.00
	Unutilized Land	0.03	0.00	0.14	0.00	0.00	0.00

**Table 8:** Changes of ecosystem service value caused by land use transformation in three geographical units from 2000 to 2018 (unit: 10,000 yuan).

2000		2018					
		Cultivated Land	Woodland	Grass Land	Waters	Construction Land	Unutilized Land
Northern Shaanxi Plateau	Cultivated land	0.00	175,581.45	93,752.08	9259.85	−29,559.98	−1255.68
	Woodland	−14,232.03	0.00	−8031.08	655.26	−10,744.65	−7291.62
	Grassland	−26,854.73	63,102.36	0.00	7479.96	−46,733.74	−8947.32
	Waters	−14,804.36	−209.99	−9112.24	0.00	−8392.93	−875.01
	Construction Land	778.96	128.90	475.27	2229.63	0.00	0.17
	Unutilized Land	9513.84	1490.66	11,787.84	2878.59	−2135.74	0.00
Guanzhong Plain	Cultivated land	0.00	7634.29	4276.24	34,293.42	−70,901.73	−866.04
	Woodland	−3486.75	0.00	−497.75	916.48	−10,419.88	−1494.06
	Grassland	−5526.27	2533.08	0.00	5842.02	−3751.46	−413.38
	Waters	−34,506.23	−1137.01	−8826.32	0.00	−6561.21	−17,690.59
	Construction Land	4027.19	412.65	322.27	459.69	0.00	41.83
	Unutilized Land	422.30	897.01	792.36	627.44	−2.25	0.00
Qinba Mountain aera	Cultivated land	0.00	26,139.82	14,324.13	17,622.82	−9995.59	−161.93
	Woodland	−13,663.72	0.00	−14,325.97	188.34	−1198.07	−578.30
	Grassland	−9853.47	21,149.56	0.00	4726.57	−1514.25	−294.43
	Waters	−4219.44	−45.15	−681.38	0.00	−214.38	−0.78
	Construction Land	298.58	4360.97	530.50	233.54	0.00	0.31
	Unutilized Land	0.85	24.16	6.76	1.25	0.00	0.00

**Table 9:** Changes in potential grain production caused by land use transformation in three geographical units between 1980 and 2018 (unit: kg/ha).

Types of Land Use Transfer	1980–2000			2000–2018		
	Northern Shaanxi Plateau	Guanzhong Plain	Qinba Mountain Aera	Northern Shaanxi Plateau	Guanzhong Plain	Qinba Mountain Aera
Cultivated land→Woodland	−50.93	−1519.76	−59.69	−934.08	−1207.01	−524.08
Cultivated land→Grassland	−28.41	−254.38	−27.25	−801.18	−4120.54	−328.64
Cultivated land→Waters	−343.89	−1468.13	−7.84	−781.64	−2949.52	−46.54
Cultivated land→Construction Land	−168.22	−512.32	−275.58	−1119.48	−1739.03	−2233.83
Cultivated land→Unutilized Land	−684.29	−67.21	−0.01	−412.28	−716.22	−28.91
Woodland→Cultivated land	77.73	1.15	250.33	217.32	10.65	577.11
Grassland→Cultivated land	295.25	923.02	34.13	639.44	1899.58	81.93
Waters→Cultivated land	441.14	765.09	138.63	1076.68	532.65	96.82
Construction Land→Cultivated land	0.21	28.91	10.22	59.28	322.63	927.39
Unutilized Land→Cultivated land	264.82	8.44	1.74	1236.51	22.80	59.06

## Data Availability

The data that support the findings of this study are available from the corresponding author upon reasonable request.
